# Risk factors for IgA nephropathy recurrence and impact on graft survival in a cohort of kidney transplanted patients

**DOI:** 10.1080/0886022X.2025.2472041

**Published:** 2025-03-06

**Authors:** Angelodaniele Napoletano, Michele Provenzano, Federica Maritati, Valeria Corradetti, Vania Cuna, Elisa Gessaroli, Chiara Abenavoli, Simona Barbuto, Marcello Demetri, Matteo Ravaioli, Giorgia Comai, Gaetano La Manna

**Affiliations:** ^a^ Nephrology, Dialysis and Kidney Transplant Unit, IRCCS Azienda Ospedaliero-Univesitaria di Bologna, Bologna, Italy; ^b^ Department of Pharmacy, Health and Nutritional Sciences, University of Calabria, Rende, CS, Italy; ^c^ Department of Medical and Surgical Sciences (DIMEC), Alma Mater Studiorum University of Bologna, Bologna, Italy; ^d^ Department of Medical and Surgical Sciences, University of Bologna, Italy; ^e^ Hepato-biliary Surgery and Transplant Unit, IRCCS Azienda Ospedaliero-Universitaria di Bologna, Bologna, Italy

**Keywords:** IgA nephropathy, kidney transplantation, IgAN recurrence, risks factors, graft survival

## Abstract

Recurrence of IgA nephropathy (IgAN) after kidney transplant (KT) appears associated with worse graft survival; thus, the identification of risk factors is worthwhile to improve pre-transplant evaluation of KT recipients and to identify the optimal treatment strategy. The aim of this study was to determine incidence, risk factors and impact on renal function and graft survival of IgAN recurrence after KT. We performed a retrospective study including 110 patients with biopsy-proven IgAN, who underwent KT at Policlinico di Sant’Orsola Hospital – University of Bologna from 2005 to 2021. IgAN recurred in 14 patients (12.7%) with a median time-to-recurrence of 59 (16–90) months. We found that a faster progression from IgAN diagnosis to end-stage kidney disease (ESKD), a younger age at ESKD, and a younger age at KT were associated with a higher risk of recurrence. During the first 2 years after KT, 24 h proteinuria was higher in patients with IgAN recurrence than in patients without (0.40 (0.11–1.8) vs 0.22 (0.18–0.37) g/day, *p* = 0.0003). During the follow-up period, a more rapid decline in eGFR was observed in the Recurrence group (*p* = 0.023). Additionally, graft survival at 10 years post-kidney transplant was significantly lower in this group (log-rank test *p* = 0.015). In conclusion, we found that patients with a more aggressive form of IgAN, who reached ESKD before 36 years of age, had an higher risk of recurrence in KT. Moreover we confirmed that recurrent IgAN, especially if clinically relevant, is associated with a worse graft outcome.

## Graphical Abstract

**Figure:**
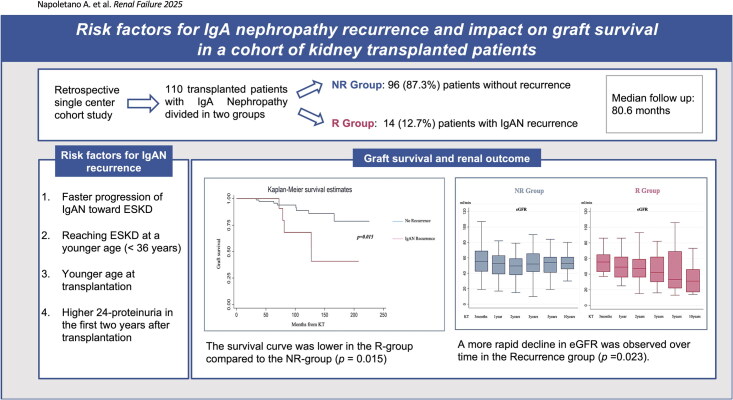

## Introduction

IgA Nephropathy (IgAN) is the most frequent primary glomerular disease worldwide, with an overall population incidence of about 2.5 cases per 100,000 [1–3]. IgA Nephropathy is an autoimmune disease that affects the kidneys, with a broad spectrum of clinical manifestations, ranging from isolated microscopic hematuria to rapidly progressive glomerulonephritis [4,5]. The epidemiological data also show that approximately 25–30% of patients with IgAN reach end-stage kidney disease (ESKD), namely the most advanced stage of kidney disease requiring renal replacement therapy, after 20 years from kidney biopsy [6]. For these patients, kidney transplant (KT) still remains the treatment of choice, but it can be complicated by the recurrence of the disease [7]. The incidence of IgAN recurrence after KT is difficult to determine due to the different graft biopsy policies among transplant centers: the highest rates are obviously reported by centers performing protocol biopsies compared to centers, which perform only for-cause biopsies [8,9]. In addition, the incidence of IgAN recurrence is influenced by the duration of follow-up, as the recurrence rate tends to increase with a longer follow-up period [10].

Currently, no well-established strategies are available for the treatment of IgAN recurrence and several studies have already shown that this condition is associated with a worse long-term graft survival [11–14]. Thus, the identification of patients at high risk for IgAN recurrence is paramount in order to improve the pre-transplant evaluation and to identify patients who need a more strictly follow up and early therapeutic strategies to slow disease progression.

This study aimed to assess the incidence of recurrent IgAN after KT in patients with ESKD due to biopsy-proven IgA Nephropathy. We also tried to identify pre and post-transplant risk factors for this condition and to analyze graft survival by comparing IgAN patients with and without disease recurrence.

## Materials and methods

### Study design and population

In this retrospective, observational, single center study, we reviewed clinical records of all patients with a histological diagnosis of IgAN on native kidneys which performed a KT at our Center (Policlinico di Sant’Orsola Hospital- University of Bologna) from January 2005 to December 2021. Inclusion criteria were: i) age > 18 years old at KT, ii) biopsy confirmed IgAN as a cause of ESKD, iii) follow up duration of at least 12 months. Exclusion criteria were: i) primary non-function, ii) recipients of multiple organ transplant.

The study protocol was approved by the ethical committee of the IRCCS Policlinico di Sant’Orsola Hospital – University of Bologna (Protocol number 586/2023/Oss/AOUBo).

### Data collection

We retrieved data about patients’ demographics, clinical history and laboratory features at time of kidney biopsy (KB), any IgAN treatment, time from KB to ESKD, type of dialysis. Data about KT characteristics and post-transplant course were also collected, including induction and maintenance immunosuppressive treatment, delayed graft function (DGF), any allograft rejection, duration of follow up, graft loss and mortality. Furthermore, laboratory tests at the time of KT and during follow up including serum creatinine, eGFR and 24 h proteinuria were collected. Patients were censored at time of graft loss or death, loss to follow up or in September 2023 (study termination).

### Definitions

Graft biopsy was performed in case of acute renal disfunction (eGFR decrease >25% or creatinine increase ≥50% from its baseline value) of unclear origin [15], persistent proteinuria > 0.5 g/day over at least 3 months or persistent microscopic hematuria of non-urological origin, donor-specific antibodies detection. All biopsies were evaluated by light microscopy and immunofluorescence. Diagnosis of IgAN recurrence was defined histologically by dominant or co-dominant IgA mesangial staining in immunofluorescence.

Diagnosis of rejection was performed on the basis of histology findings and was scored retrospectively according to the recently revised Banff classification [16].

Delayed graft function was defined as the use of dialysis within seven days of the KT [17].

Graft loss was defined as resuming chronic dialysis or new KT.

### Statistical analysis

Statistical analyses were performed using STATA, version 16 (Stata Corp. College Station, TX, USA).

Continuous variables were reported as mean and standard deviation (SD) or median and interquartile range (IQR) according to distribution. Categorical variables were depicted as number (n) and percentage (%) and compared across different groups using contingency tables and Fisher’s exact test. Comparisons between groups (Recurrence, R-group vs No-Recurrence, NR-group) in terms of characteristics were assessed by means of Student’s *t* test or Kruskal–Wallis test for continuous variables, according to distribution, and chi-squared test for categorical variables. Univariate analysis for the association between pre-transplant and post-transplant characteristics and IgAN recurrence were tested by Cox proportional hazard regression analysis, varying the follow-up used. In the first case, interval between KB and IgAN recurrence was used. In the second case, interval between KT and graft failure was considered. Due to the long follow-up, proportional hazard assumption was verified with Schoenfeld residuals (data not shown). Unadjusted risk of graft failure among R and NR groups was tested with Kaplan–Meier analysis and log-rank test. Cox proportional hazard model was also used to assess, in multivariable analysis, the role of IgA recurrence as predictor of graft failure after KT, after adjusting for the main confounders. To this aim, the confounders (rejection and eGFR maximum levels within 3 months after KT) have been included into the Cox model using an “*a priori*” method. A two-sided *p*-value ≤ 0.05 was considered to indicate statistical significance. The receiver operating characteristic (ROC) curves were used to evaluate the variable’s ability for classifying IgAN recurrence status, which was, younger versus older age at ESKD. To find a cut-point that maximizes the variable’s differentiating ability from the ROC curves the Youden index (J) was computed. J was defined as the variable’s value for which equal weight was given to sensitivity and specificity [18,19].

## Results

### Patients and graft characteristics

From 1 January 2005 to 31 December 2021, a total of 1449 KT were performed at our Center, 126 (8.7%) of them in patients with biopsy-proven IgAN. 16 of these patients were excluded from the statistical analysis (10 patients were lost to follow-up within 12 months from KT and 6 patients had a primary non-function). The main demographic and clinical characteristics of the 110 patients included in the study are summarized in Table 1. In our cohort, there was a higher prevalence of male sex (82.7%) and the mean age at the time of KT was 47.5 ± 12.6 years. 11 patients (10.0%) had already received one previous KT. Overall, 78 patients (70.9%) had received a KT from a deceased donor, whereas 32 from a living donor. The mean age of the donors was 53.3 ± 15.2 years. Induction therapy consisted in basiliximab in the majority of patients (85, 77.2%) and the main maintenance therapy was based on calcineurin inhibitor associated with mycophenolic acid and prednisone (91, 82.7%). 32 patients (29.1%) experienced DGF, while 12 patients (10.9%) had at least one episode of histologically proven rejection. Over a median follow-up of 80.6 (47.7–134.0) months, the loss of the transplanted organ occurred in 13 patients (11.8%) and 9 patients (8.2%) died with a functioning graft.

**Table 1.** Pre and post-transplant characteristics of the 110 KT patients with kidney failure due to biopsy-proven IgAN.

	Overall (*n* = 110)	NR group (*n* = 96)	R group (*n* = 14)	*P*
Male gender, *%*	82.7	81.2	92.9	0.283
**Pre-transplant**				
Characteristics at IgAN diagnosis				
Age, *years*	31.1 ± 11.5	31.2 ± 11.1	29.9 ± 14.4	0.690
eGFR, mL/min/1.73m^2^	58.5 ± 34.5	58.3 ± 33.5	60.2 ± 41.9	0.857
24 h proteinuria, *g/day*	1.9 [0.9–3.5]	2.0 [0.8–3.5]	1.5 [1.1–3.4]	0.865
24 h proteinuria >1g/day, *%*	68.8	73.1	84.6	0.382
Macrohematuria, *%*	39.6	39.1	42.9	0.789
Microhematuria, %	60.4	60.9	57.1	–
Hypertension, (*yes* vs. *no)* %	63.2	63.0	64.3	0.928
Therapy for IgAN				
RAASi, (*yes vs. no)* %	80.4	79.8	84.6	0.682
Immunosuppressive agents, (*yes vs. no) %*	67.3	68.3	61.5	0.627
Age at ESKD, *years*	**42.9 ± 12.4**	**43.8 ± 12.2**	**36.4 ± 12.3**	**0.036**
Time from KB to ESKD, *months*	**120 [54–212]**	**141 [58–231]**	**77 [13–139]**	**0.023**
Pre-emptive KT, *%*	9.1	10.4	0.0	0.205
Type of renal replacement therapy				0.500
Peritoneal dialysis, *%*	20.0	20.8	14.3	
Hemodialysis + Peritoneal dialysis, *%*	14.5	14.5	14.3	
Hemodialysis, *%*	56.3	54.1	71.4	
**Post-transplant**				
Recipient age at KT, *years*	**47.5 ± 12.6**	**48.7 ± 12.1**	**39.9 ± 13.3**	**0.014**
Donor age, *years*	53.3 ± 15.2	53.8 ± 15.0	49.3 ± 16.3	0.296
Living donor kidney transplant, %	29.1	27.1	42.9	0.225
Retransplantation, %	10.0	11.4	0.0	0.182
Number of HLA A/B/DR mismatches	3.5 **±** 1.3	3.5 **±** 1.3	3.6 **±** 1.4	0.752
Induction therapy with ATG (vs BSX), %	22.7	21.8	28.5	0.576
Maintenance immunosuppressive therapy				0.614
CNI + Antimetabolite + steroid, %	82.7	82.9	85.7	
CNI + mTORi + steroid, %	13.6	14.5	7.14	
Steroid free, %	0.9	1.0	0.00	
CNI + steroid, %	2.73	2.08	7.14	
DGF, *%*	29.1	31.2	14.3	0.192
De novo DSA, %	7.95	6.5	18.2	0.180
Rejection (ABMR+ TCMR), *%*	10.9	9.4	21.4	0.177

Values are presented as Number (%), Mean ± SD, or Median (IQR).

eGFR: estimated glomerular filtration rate; RAASi: Renin-Angiotensin-Aldosterone System inhibitors; ESKD: end stage kidney disease; KB: kidney biopsy; KT: kidney transplant; ATG: anti-thymocyte globulin; BSX: Basiliximab; CNI: calcineurin inhibitors; DGF: delayed graft function; DSA: donor-specific antibodies; ABMR: antibody-mediated rejection; TCMR: T-cell-mediated rejection.

### IgAN recurrence

14/110 patients (12.7%) had biopsy-proven IgAN recurrence. The recurrence occurred at a median time of 59 (16–90) months after KT. At the time of recurrence, the mean eGFR was 42 ± 22 mL/min per 1.73 m^2^, the median proteinuria was 1.9 (1.4–3.0) g/day, and microhematuria was present in 11 patients (78.6%). Out of a total of 110 patients in our cohort, 35 underwent at least one renal transplant biopsy. The clinical indications for graft biopsy in this subgroup of patients were: proteinuria >0.5 g/day with or without a worsening of serum creatinine in 18 instances (51.4%), a rise in serum creatinine in 16 (45.7%) patients and persistent microhematuria in 1 (2.9%). When considering only these 35 patients and excluding the remaining 75 who did not undergo a biopsy, the recurrence rate of IgA nephropathy increased to 40.0% (14/35 patients).

### Risk factors for IgAN recurrence

**Figure 1.**
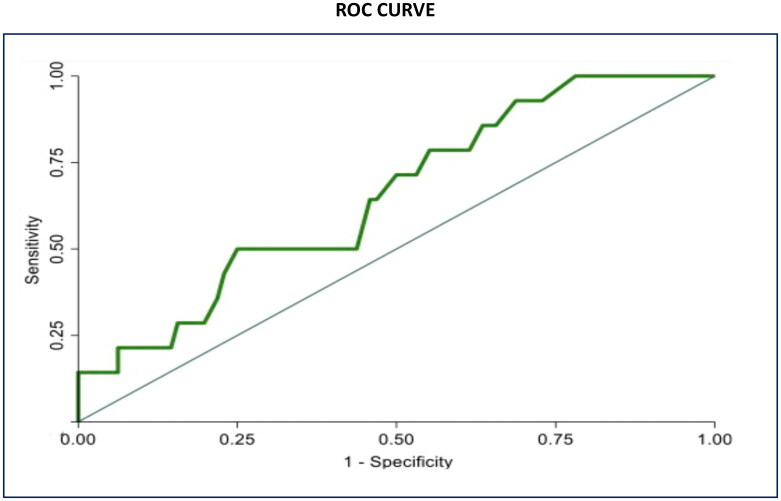
Receiver operating characteristic curve (ROC) for the association of age at ESKD and IgA recurrence. Area under the curve reported a good discrimination of age at ESKD on the recurrence of IgA nephropathy.

Considering pre-transplant variables, there was no statistically significant difference among the R-group and NR-group in terms of age, eGFR, 24h-proteinuria and hematuria at time of kidney biopsy and history for hypertension. In addition, no significant difference was found in IgAN therapy and modality of dialysis treatment. Patients who had IgAN recurrence reached ESKD at a younger age compared to patients without recurrence (36.4 ± 12.3 vs 43.8 ± 12.2 years, *p* = 0.036), and they were characterized by a shorter time from histological diagnosis of IgAN to ESKD (77 (13–139) vs 141 (58–231) months, *p* = 0.023). Moreover, patients with post-transplant IgAN recurrence were significantly younger at the time of KT (39.9 ± 13.3 vs 48.7 ± 12.1 years, *p* = 0.014). No statistically significant differences emerged between the two groups in terms of donor characteristics, HLA mismatches, immunosuppressive therapy, re-transplantation rate, rejection episodes, *de novo* DSA and DGF (Table 1). These data were confirmed at univariate analysis (Table 2), which identified as significant predictors for recurrent IgAN: the age at ESKD (HR 0.95, 95% CI 0.90–0.99; *p* 0.016), the time from IgAN diagnosis to ESKD (HR 0.87, 95% CI 0.79–0.94; *p* 0.001) and the recipient age at KT (HR 0.92, 95% CI 0.87–0.96; *p* 0.001). Moreover, ROC curve analysis, aiming at identifying the cutoff of age at ESKD below which the prediction of IgA recurrence is optimal found that patients reaching ESKD at 36 year-old age or earlier had the highest risk of recurrence (Figure 1).

**Table 2.** The hazard ratios of risk factors for IgAN recurrence.

	Hazard ratio	95% Confidence interval	*p*
Age at ESKD	0.95	0.90–0.99	0.016
Time from KB to ESKD	0.87	0.79–0.94	0.001
Recipient age at KT	0.92	0.87–0.96	0.001

ESKD: End-stage kidney disease; KB: Kidney biopsy.

### Renal outcome and graft survival

**Figure 2.**
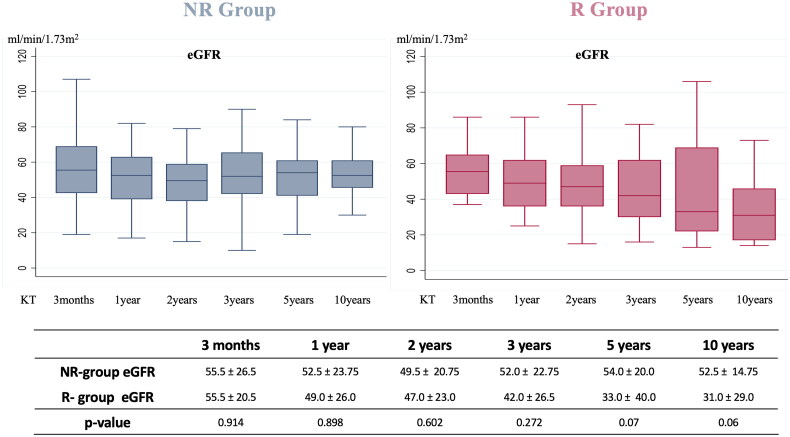
Renal function, shown as median eGFR, was similar in the two groups during the first 3 years. However, from the 5th year the recurrence group showed a more pronounced decline in eGFR. The difference in the eGFR trend over time between the two groups was statistically significant (linear mixed model *p* = 0.023).

**Figure 3.**
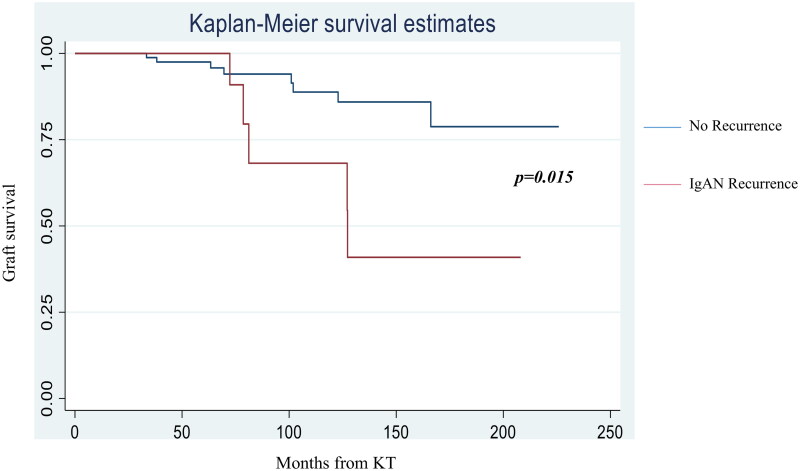
Kaplan–Meier graft survival curves. *R*-group survival curve was significantly lower than NR group curve (log rank test *p* = 0.015).

The median follow-up was similar for patients with and without IgAN recurrence, 79.9 (72.3–127.3) and 80.6 (44.0–135.7) months, *p* = 0.572). The median value of 24 h-proteinuria within two years from KT, was significantly higher in patients with recurrence than in patients without (0.40 (0.11–1.8) vs 0.22 (0.18–0.37) g/day, *p* = 0.0003). Additionally the rate of persistent microhematuria was significantly higher in the R group compared to the NR group (78.5% vs 28.1%, *p* < 0.05). The highest eGFR value within the first three months after KT did not differ among the two groups. As shown in Figure 2, during the first 3 years after KT, renal function was similar between the two groups. Starting from the fifth year, a more pronounced downward trend in eGFR was observed in the recurrence group, even though the difference in median eGFR, between the groups, was not statistically significant (median eGFR at 5 years was 33.0 ± 40.0 mL/min per 1.73 m^2^ in patients with recurrence and 54.0 ± 20.0 mL/min per 1.73 m^2^ in patients without recurrence, *p* = 0.07). However the comparison of eGFR curves by using the mixed linear model demonstrated a significant difference in the eGFR trend over time in the two groups (*p* = 0.023). The analysis of data about graft survival showed that five (35.7%) patients in IgAN recurrence group and just eight (8.3%, *p* = 0.010) patients without recurrence experienced graft loss during the follow up, with a median time to graft loss of 80.0 (77.0–125.0) and 83.5 (49.5–110.5) months, respectively (*p* = 0.380). Moreover, in order to assess the impact of IgAN recurrence on graft failure, a multivariate analysis was conducted, including other predictors of graft loss (rejection and highest eGFR value within 3 months from KT). The data confirmed that IgAN recurrence was a predictive factor for graft loss (HR 3.56, 90% CI 1.16–10.9; *p* 0.027). Furthermore, a statistically significant difference was found in the Kaplan–Meier curves for death-censored graft survival among the two groups (log-rank test *p* = 0.015) and 10 years after KT, the graft survival was 67% in patients who developed post-transplant IgAN recurrence, versus 87% in those who did not (Figure 3).

## Discussion

The recurrence of IgAN after kidney transplantation occurs in 10 to 60% of patients and it seems to be associated with a worse graft survival [10,20,21]. In this single-center study including 110 kidney-transplanted patients with ESKD due to biopsy proven IgAN, over a median follow-up of 80.6 (47.7–134.0) months, we found a recurrence rate of 12.7%. Disease recurrence occurred at a median of 59 (16–90) months after transplantation. The low rate of recurrence in the current study can be explained by the fact that protocol biopsies are not performed in our center. As a matter of fact, biopsies were performed because of abnormal urinalysis or serum creatinine worsening, with the most frequent indication represented by persistent proteinuria > 0.5 g/day (51.4%). However, if we consider only patients who underwent at least one KT biopsy the IgAN recurrence incidence rises to 40%. These results suggest that the true recurrence rate of IgAN could be higher if all patients in the cohort had undergone at least one kidney biopsy and support the findings reported by Uffing et al. in a large multicenter study [22]. In this study, we tried to assess pre-transplant and post-transplant risk factors for IgAN recurrence by comparing clinical features of patients with and without recurrence. The analysis of pre-transplant features of R group and NR group showed that patients with recurrence, compared to patients without recurrence, were characterized by a faster progression from diagnosis to ESKD (77 (13–139) vs 141 (58–231) months, *p* = 0.023). These findings are consistent with those reported in in a recent meta-analysis, where the authors found that patients with a more rapid progression of the primary disease were at higher risk of recurrence [23]. Interestingly we found also that patients with recurrence reached ESKD at a younger age (36.4 ± 12.3 vs 43.8 ± 12.2 years old, *p* = 0.036). Both the rapidity of progression of IgAN toward ESKD and the achievement of ESKD at a younger age may be an expression of the “aggressiveness” of the disease and therefore should be taken into consideration to assess the risk of IgAN recurrence in the recipients. With ROC analysis we determined a possible cut-off value of age at ESKD, which could be used for risk stratification of IgAN recurrence. We found that reaching ESKD before the age of 36 years, was associated with an increased risk of recurrence in KT. The other variables collected at the time of kidney biopsy on native kidneys, such as eGFR, 24 h proteinuria, the presence of hypertension or micro/macrohematuria, did not differ in the two groups. In a recent study from the Columbia University, Avasare et al. demonstrated that the presence of crescents (cellular or fibrocellular) on native kidneys biopsies was associated with a greater risk of recurrence in the transplanted kidney, suggesting the idea that a more active form of IgAN had a higher possibility to recur in the transplant [24]. Given the absence of comprehensive histological data and MEST-C scoring for native kidney biopsies in a significant number of patients in our cohort, we were unable to assess the correlation between histological findings and the risk of IgAN recurrence. At the time of transplantation, the only significant difference between the two groups was represented by the age of the recipients, which appeared significantly lower in the recurrence group (39.9 ± 13.3 vs 48.7 ± 12.1 years, *p* = 0.014). This result is in line with data already present in the literature [25,26]; in a recent meta-analysis about risk factors for IgAN recurrence, the authors argued that the stronger immune system makes young patients more inclined to recurrence [27]. In our cohort, we did not observe any significant difference in the number of HLA mismatches between R-group and NR-group. In the multicenter TANGO study, the authors found a higher incidence of IgAN recurrence in patients with preformed DSA before kidney transplantation, as well as in those who developed *de novo* DSA during the follow-up period. In our study, we were unable to assess the impact of preformed DSA, as none of the patients had significant preexisting DSA at the time of kidney transplantation. Additionally, we did not detect a significant difference in the development of *de novo* DSA between the group with and without recurrence. However, it is important to note that the small sample size of our population limits the ability to draw meaningful conclusions. The authors of the TANGO study suggested that both IgA nephropathy recurrence and *de novo* DSA could be related to insufficient immunosuppression in the patient. However, this correlation requires further investigation in future studies [22]. Published studies reported that transplantation from living donor was associated with a higher risk of the recurrence of IgAN [13,28]. In our study, we did not find differences in the type of donor (living or deceased) in patients with and without recurrence, similarly to what Moroni et al. found in a study on 190 transplanted patients affected by IgAN [11]. Interestingly in our cohort, re-transplantation was not associated with a higher risk of recurrence and none of the patients in the recurrence group had received a previous KT. The role of induction and maintenance immunosuppressive therapy in the recurrent IgAN has yet to be defined [29,30]. In some previous studies the early steroid withdrawal was associated with a greater incidence of recurrence, but other studies failed to confirm this association [31–35]. However, it should be considered that in our population, only one patient had a steroid-free maintenance therapy; for this reason, in our cohort it wasn’t possible to assess the impact of early suspension of steroid therapy on recurrence risk. In a recent meta- analysis, Li Y et al. found that patients who received induction therapy with IL-2R antibodies had a lower risk of recurrence; in contrast, no differences were observed in induction or maintenance therapies between the two groups in our cohort [23]. Consistent with previous studies, we observed a higher value of 24-proteinuria, in particular in the first two years post-KT, in the group with recurrence [26]. A recent Swiss study showed that proteinuria values in patients with IgAN recurrence were higher than those in patients without recurrence in the post-transplant period [36]. These findings provide additional evidence that proteinuria is a reliable and early marker of glomerular damage and should be strictly monitored over time in these patients. In fact, even a slight, but progressive, increase in proteinuria could be a sign of recurrent IgAN. We also found a significant difference in the rate of persistent microhematuria between patients with recurrence (78.5%) and those without (28.1%). In a study including 112 patients with IgAN, Sevillano M. et al. showed that a more severe magnitude of hematuria was associated with a higher grade of histological lesions, particularly mesangial hypercellularity, in kidney biopsies. For this reason, hematuria can be considered a marker of IgAN activity and should be properly evaluated during the follow-up of these patients [37]. In the evaluation of renal outcome, we analyzed the trend of kidney function over time in the two groups, by comparing the median values of eGFR at each follow up visit. Despite the highest eGFR reached within 3 months from transplantation was similar in the two groups, our study showed that starting from the 5th year the trajectories of eGFR curves tended to diverge. In fact, there was a significant difference in the eGFR trend over time in the two groups (*p* = 0.023), with a more pronounced deterioration of renal function in the group with recurrence. Jäger C. et al. documented how starting from the seventh year after KT, the value of serum creatinine in patients with recurrence was significantly higher than in patients without recurrence [36]. Similarly, Choy Y. et al. found that starting from the eighth year after transplantation, patients with recurrence had a more rapid decline of renal function [12]. In a recent American study that included 100 patients with biopsy-proven recurrent IgAN, the authors demonstrated the prognostic usefulness of the MEST-C score for allograft biopsies. This multicenter study showed that a higher MEST-C score in recurrent IgAN was associated with reduced graft survival. In our cohort, due to the small number of patients with recurrence, it was not possible to establish a meaningful correlation between histological findings on allograft biopsies and graft survival [38]. Treatment of recurrent IgAN in our cohort was mainly represented by ACEi or ARBs, with or without systemic corticosteroid therapy. Just in one case targeted-release formulation of budesonide was prescribed. Moreover, studies with long term follow up reported that recurrent IgAN had a negative impact on graft survival [11,39]. In our population, the graft loss rate in patients with recurrence was approximately 4 times higher than in patients without recurrence (35.7% vs 8.3%, *p* = 0.01). We also found that the 10-year graft survival rate was significantly lower in patients with recurrence compared to patients without recurrence. These findings support the notion that IgAN recurrence can adversely impact long-term graft survival, as previously documented in the TANGO study. Specifically, Uffing et al. reported that recurrence of IgA nephropathy was associated with a 3.7-fold increased risk of graft loss, emphasizing the detrimental impact of this disease on graft outcomes [22].

In conclusion, our study showed that IgAN recurrence has a negative impact on renal outcome and graft survival. Since protocol biopsies are not performed in our center, the findings reported in the current study can be valid for recurrent IgAN with clinical signs rather than for asymptomatic IgA deposition. Conversely patients without IgAN recurrence or a silent recurrence (without clinical signs) had a good graft survival.

The main limitation of our study is represented by its retrospective design; additionally, since detailed data about native kidney biopsies were not available we were unable to analyze the impact of histological findings on the risk of recurrence. Despite the relatively small number of the patients included in the analysis, we were able to identify several risk factors for IgAN recurrence, that could be considered to estimate the likelihood of this adverse event after kidney transplantation.

In particular, we found that a younger age at ESKD, a more rapid progression of IgAN toward ESKD and a younger age at KT were significantly associated with an augmented risk for recurrence.

Notably, we observed that reaching ESKD before the age of 36, in patients with IgAN, is associated with a higher risk of disease recurrence after KT. These findings suggest that a more aggressive form of IgAN can recur more frequently after KT.

A detailed collection of anamnestic, clinical and if possible histological data about IgAN could be important to stratify the risk of recurrence in candidates for KT, in order to define surveillance and therapeutic strategies to improve graft survival.

## Data Availability

The datasets generated during and/or analyzed during the current study are available from the corresponding author on reasonable request

## References

[1] Storrar J, Chinnadurai R, Sinha S, et al. The epidemiology and evolution of IgA nephropathy over two decades: a single centre experience. PLoS One. 2022;17(9):e0268421. doi: 10.1371/journal.pone.0268421. 36048745 PMC9436111

[2] Pattrapornpisut P, Avila-Casado C, Reich HN. IgA Nephropathy: core curriculum 2021. Am J Kidney Dis. 2021;78(3):429–441. doi: 10.1053/j.ajkd.2021.01.024. 34247883

[3] McGrogan A, Franssen CFM, De Vries CS. The incidence of primary glomerulonephritis worldwide: a systematic review of the literature. Nephrol Dial Transplant. 2011;26(2):414–430. doi: 10.1093/ndt/gfq665. 21068142

[4] KDIGO 2021 Clinical Practice Guideline for the Management of Glomerular Diseases. Kidney Int. 2021;100(4S):S1–S276. 34556256 10.1016/j.kint.2021.05.021

[5] Lai KN, Tang SCW, Schena FP, et al. IgA nephropathy. Nat Rev Dis Primers. 2016;2(1):16001. doi: 10.1038/nrdp.2016.1. 27189177

[6] Moriyama T, Tanaka K, Iwasaki C, et al. Prognosis in IgA nephropathy: 30-Year analysis of 1,012 patients at a single center in Japan. PLoS One. 2014;9(3):e91756. Mar. doi: 10.1371/journal.pone.0091756. 24658533 PMC3962373

[7] Berger J, Noël LH, Nabarra B. Recurrence of mesangial IgA nephropathy after renal transplantation. Contrib Nephrol. 1984;40:195–197. doi: 10.1159/000409749. 6389000

[8] Sofue T, Suzuki H, Ueda N, et al. Post-transplant immunoglobulin A deposition and nephropathy in allografts. Nephrology (Carlton). 2018;23 Suppl 2(S2):4–9. doi: 10.1111/nep.13281. 29968406

[9] Wyld ML, Chadban SJ. Recurrent IgA nephropathy after kidney transplantation. Transplantation. 2016;100(9):1827–1832. doi: 10.1097/TP.0000000000001093. 26895219

[10] Allen PJ, Chadban SJ, Craig JC, et al. Recurrent glomerulonephritis after kidney transplantation: risk factors and allograft outcomes. Kidney Int. 2017;92(2):461–469. doi: 10.1016/j.kint.2017.03.015. 28601198

[11] Moroni G, Longhi S, Quaglini S, et al. The long-term outcome of renal transplantation of IgA nephropathy and the impact of recurrence on graft survival. Nephrol Dial Transplant. 2013;28(5):1305–1314. doi: 10.1093/ndt/gfs472. 23229925

[12] Choy BY, Chan TM, Lo SK, et al. Renal transplantation in patients with primary immunoglobulin A nephropathy. Nephrol Dial Transplant. 2003;18(11):2399–2404. doi: 10.1093/ndt/gfg373. 14551373

[13] Han SS, Huh W, Park SK, et al. Impact of recurrent disease and chronic allograft nephropathy on the long-term allograft outcome in patients with IgA nephropathy. Transpl Int. 2010;23(2):169–175. doi: 10.1111/j.1432-2277.2009.00966.x. 19761553

[14] Floege J, Gröne HJ. Recurrent IgA nephropathy in the renal allograft: not a benign condition. Nephrol Dial Transplant. 2013;28(5):1070–1073. doi: 10.1093/ndt/gft077. 23674835

[15] Bellomo R, Ronco C, Kellum JA, et al. Acute renal failure – definition, outcome measures, animal models, fluid therapy and information technology needs: the Second International Consensus Conference of the Acute Dialysis Quality Initiative (ADQI) Group. Crit Care. 2004;8(4):R204–12. Epub 2004 May 24. PMID: 15312219; PMCID: PMC522841. doi: 10.1186/cc2872. 15312219 PMC522841

[16] Haas M, Loupy A, Lefaucheur C, et al. The Banff 2017 Kidney Meeting Report: revised diagnostic criteria for chronic active T cell-mediated rejection, antibody-mediated rejection, and prospects for integrative ­endpoints for next-generation clinical trials. Am J Transplant. 2018;18(2):293–307. doi: 10.1111/ajt.14625. 29243394 PMC5817248

[17] Rolak S, Djamali A, Mandelbrot DA, et al. Outcomes of delayed graft function in kidney transplant recipients stratified by histologic biopsy findings. Transplant Proc. 2021;53(5):1462–1469. Epub 2021 Feb 10. PMID: 33579551. doi: 10.1016/j.transproceed.2021.01.012. 33579551

[18] DeLong ER, DeLong DM, Clarke-Pearson DL. Comparing the areas under two or more correlated receiver operating characteristic curves: a nonparametric approach. Biometrics. 1988;44(3):837–845. 3203132

[19] Ruopp MD, Perkins NJ, Whitcomb BW, et al. Youden Index and optimal cutpoint estimated from observations affected by a lower limit of detection. Biom J. 2008;50(3):419–430. doi: 10.1002/bimj.200710415.4. 18435502 PMC2515362

[20] Han HS, Lubetzky ML, Anandasivam NS, et al. Recurrent immunoglobulin A Nephropathy after kidney transplant—an updated review. Transplantology. 2023;4(3):161–177. doi: 10.3390/transplantology4030016.

[21] Lionaki S, Makropoulos I, Panagiotellis K, et al. Kidney transplantation outcomes in patients with IgA nephropathy and other glomerular and non-glomerular primary diseases in the new era of immunosuppression. PLoS One. 2021;16(8):e0253337. doi: 10.1371/journal.pone.0253337. 34403416 PMC8370606

[22] Uffing A, Pérez-Saéz MJ, Jouve T, et al. Recurrence of IgA Nephropathy after kidney transplantation in adults. Clin J Am Soc Nephrol. 2021;16(8):1247–1255. doi: 10.2215/CJN.00910121. PMID: 34362788; PMCID: PMC8455056. 34362788 PMC8455056

[23] Li Y, Tang Y, Lin T, et al. Risk factors and outcomes of IgA nephropathy recurrence after kidney transplantation: a systematic review and meta-analysis. Front Immunol. 2023;14:1277017. doi: 10.3389/fimmu.2023.1277017. 38090563 PMC10713786

[24] Avasare RS, Rosenstiel PE, Zaky ZS, et al. Predicting post-transplant recurrence of IgA nephropathy: the importance of crescents. Am J Nephrol. 2017;45(2):99–106. doi: 10.1159/000453081. 28056461 PMC5296401

[25] Maixnerova D, Hruba P, Neprasova M, et al. Outcome of 313 Czech patients with IgA nephropathy after renal transplantation. Front Immunol. 2021;12:726215. doi: 10.3389/fimmu.2021.726215. 34659212 PMC8515028

[26] Ponticelli C, Traversi L, Feliciani A, et al. Kidney transplantation in patients with IgA mesangial glomerulonephritis. Kidney Int. 2001;60(5):1948–1954. doi: 10.1046/j.1523-1755.2001.00006.x. 11703614

[27] Bai J, Wu Q, Chen J, et al. Risk factors for recurrent IgA nephropathy after renal transplantation: a meta-analysis. Biomol Biomed. 2023;23(3):364–375. doi: 10.17305/bjbms.2022.8369. 36475355 PMC10171446

[28] McDonald SP, Russ GR. Recurrence of IgA nephropathy among renal allograft recipients from living donors is greater among those with zero HLA mismatches. Transplantation. 2006;82(6):759–762. doi: 10.1097/01.tp.0000230131.66971.45. 17006322

[29] Berthoux F, El Deeb S, Mariat C, et al. Antithymocyte globulin (ATG) induction therapy and disease recurrence in renal transplant recipients with primary IGA nephropathy. Transplantation. 2008;85(10):1505–1507. doi: 10.1097/TP.0b013e3181705ad4. 18497694

[30] Lee KW, Kim KS, Lee JS, et al. Impact of induction ­immunosuppression on the recurrence of primary IgA nephropathy. Transplant Proc. 2019;51(5):1491–1495. doi: 10.1016/j.transproceed.2019.01.115. 31010698

[31] Cazorla-López JM, Wu J, Villanego-Fernández F, et al. IgA Nephropathy after renal transplant: recurrences and de novo cases. Transplant Proc. 2020;52(2):515–518. doi: 10.1016/j.transproceed.2019.12.008. 32037064

[32] Leeaphorn N, Garg N, Khankin EV, et al. Recurrence of IgA nephropathy after kidney transplantation in ­steroid continuation versus early steroid-withdrawal regimens: a retrospective analysis of the UNOS/OPTN database. Transpl Int. 2018;31(2):175–186. doi: 10.1111/tri.13075. 28926143 PMC5762402

[33] Von Visger JR, Gunay Y, Andreoni KA, et al. The risk of recurrent IgA nephropathy in a steroid-free protocol and other modifying immunosuppression. Clin Transplant. 2014;28(8):845–854. doi: 10.1111/ctr.12389. 24869763

[34] Clayton P, McDonald S, Chadban S. Steroids and recurrent IgA nephropathy after kidney transplantation. Am J Transplant. 2011;11(8):1645–1649. doi: 10.1111/j.1600-6143.2011.03667.x. 21797974

[35] Uffing A, Pérez-Sáez MJ, La Manna G, et al. A large, international study on post-transplant glomerular diseases: the TANGO project. BMC Nephrol. 2018;19(1):229. doi: 10.1186/s12882-018-1025-z. PMC6136179 30208881

[36] Jäger C, Stampf S, Molyneux K, et al. Recurrence of IgA nephropathy after kidney transplantation: experience from the Swiss transplant cohort study. BMC Nephrol. 2022;23(1):178. doi: 10.1186/s12882-022-02802-x. PMC9088042 35538438

[37] Sevillano AM, Gutiérrez E, Yuste C, et al. Remission of hematuria improves renal survival in IgA Nephropathy. J Am Soc Nephrol. 2017;28(10):3089–3099. doi: 10.1681/ASN.2017010108. 28592423 PMC5619972

[38] Alachkar N, Delsante M, Greenberg RS, et al. Evaluation of the Modified Oxford Score in recurrent IgA Nephropathy in North American Kidney Transplant ­recipients: the Banff Recurrent Glomerulonephritis Working Group Report. Transplantation. 2023;107(9):2055–2063. doi: 10.1097/TP.0000000000004640. 37202854

[39] Kavanagh CR, Zanoni F, Leal R, et al. Clinical predictors and prognosis of recurrent IgA nephropathy in the kidney allograft. Glomerular Dis. 2022;2(1):42–53. doi: 10.1159/000519834. 35450416 PMC9017582

